# Non-insidious Large Joint Manifestation of Severe Cachectic Rheumatoid Arthritis

**DOI:** 10.7759/cureus.3266

**Published:** 2018-09-07

**Authors:** Simcha Weissman, Mira Alsheikh, Khalil Kamar, Joshua Breslin, Anthony Khabut, Mohammad G Maruf

**Affiliations:** 1 Medical Student, Touro College of Osteopathic Medicine, New York, USA; 2 Internal Medicine Resident, Staten Island University Hospital, Northwell Health, Staten Island, USA; 3 Medicine, Staten Island University Hospital, Staten Island, USA; 4 Medicine, Staten Island University Hospital, Roslyn, USA

**Keywords:** arthocentesis, pneumothorax, cachexia, rheumatoid arthritis

## Abstract

Rheumatoid arthritis (RA) is an autoimmune disorder in which constitutional symptoms typically occur before joint swelling becomes a true clinical phenomenon. Weight loss, although common, is generally mild in nature and occurs after long standing inflammation. While large joints do become inflamed, RA has a much stronger predilection for the small joints. Our case is a rarity in the fact that there was initial large joint swelling without long standing inflammation. Additionally, the weight loss was cachectic in nature and extreme. Furthermore, while extra-articular manifestations do commonly occur, spontaneous pneumothorax is certainly atypical. The content of this manuscript serves to enlighten hospitalist physicians and residents, as to the odd way in which a case like this may present.

## Introduction

Rheumatoid arthritis (RA) is a chronic, debilitating, and destructive inflammatory disease of which an exact etiology is currently unknown. Like many autoimmune disorders, an external environmental trigger such as cigarette smoking, infection, or trauma is needed to set off the inflammatory reaction in already genetically susceptible individuals. Two distinct HLA–DRβ alleles, Dw4 and Dw14, were found in RA patients suggesting that these two alleles are susceptibility genes [1]. In the majority of cases the inflammation reaction begins with the small joints of the hands and feet namely the proximal-inter-pharyngeal joint (PIP), metacarpal-pharyngeal (MCP), and carpal-metacarpal (CMC) joints—with obvious sparing of the distal-inter-pharyngeal (DIP) joints—before attacking the larger joints such as the knees and shoulders. In addition, most patients with RA have an onset insidious in nature, taking months of high inflammatory states, often beginning with fever, malaise, and weakness, before progressing to actual joint swelling. Weight loss is common in any inflammatory condition but it is generally mild and correlated with late progressive disease. We present a rare case of systemic RA presenting initially with large joint involvement and rapid weight loss, not preceded by periods of insidious constitutional symptoms.

## Case presentation

Our patient is a 51-year-old African American male who presented to the emergency department (ED) with a chief complaint of bilateral knee pain and weight loss. Upon interviewing the patient, he admitted to a weight loss of 52 pounds, all of which had occurred over the last eight weeks. Around this same time he recalls having trauma to his knees while colliding with his opponent during a game of basketball. Beginning in this same eight week period, he has had horrible knee pain, making it increasingly difficult for him to ambulate. Upon admission he was tachycardic with a heart rate of 127 bpm, afebrile with a temperature of 99.0 F, and his blood pressure was 124/63 mmHg. On physical exam, his knees were stiff, moderately swollen, moderately erythematous, and were extremely tender to palpation both medially and laterally along the joint line. He appeared cachectic, alert, oriented, and his mucous membranes were moist. He also happened to be tall and slender of habitus. His cardiovascular, pulmonary, abdominal, and genitourinary system exam findings were benign. He denied any shortness of breath, chest pain, melena, abdominal pain, night sweats, fever, chills, or changes in bowel movements. He denied any usage of drugs, tobacco, or alcohol. He denied ever having a colonoscopy. The patient had no pertinent past medical, surgical, or family history, although he admitted to not seeing a doctor since his teenage years. His laboratory results were as follows: white blood cell count (WBC) of 18.84, hemoglobin (Hg) of 8.4, and mean corpuscular volume (MCV) of 76.7. Knee X-rays done in the ED ruled out any acute fractures.

On the medicine floors, an extensive laboratory workup was ordered keeping infectious, malignancy, and rheumatologic issues on the differential. His erythrocyte sedimentation rate (ESR) and C-reactive protein (CRP) both came back elevated at 105 and 10.67, respectively, displaying severe inflammation. His WBC count was 23.25. His C3/C4 complement levels were normal at 157 and 33, respectively. His cytoplasmic antineutrophil cytoplasmic antibodies (c-ANCA) and perinuclear antineutrophil cytoplasmic antibodies (p-ANCA) were also negative. A drug panel came back negative. His Lyme serology, human immunodeficiency virus (HIV), syphilis, hepatitis A, B, and C panels were all negative as well. His antinuclear antibodies (ANA) titer was negative, while his double stranded DNA antibody (Ds-DNA) was mildly elevated at 51. The patient was started on 400 mg Ibuprofen PRN, which didn’t yield clinical improvement. Iron studies were ordered and came back consistent with anemia of chronic disease, likely explaining his tachycardia, with a serum ferritin of 613 and total iron binding capacity of 143. His thyroid workup came back normal with a thyroid-stimulating hormone (TSH) value of 3.23.

On hospital day three, a rheumatology consult was placed to perform an arthrocentesis of the knee joint. The tap came back showing a cell count of 39,346 and was negative for crystals, ruling out both septic arthritis and gout. The patient was started on 10 mg prednisone BID, which yielded much improvement clinically. The swelling minimally decreased and the stiffness receded. The patient was then able to ambulate with the help of physical therapy (PT). The patient refused a fecal occult blood test (FOBT) but agreed that he would have one done outpatient.

**Figure 1:**
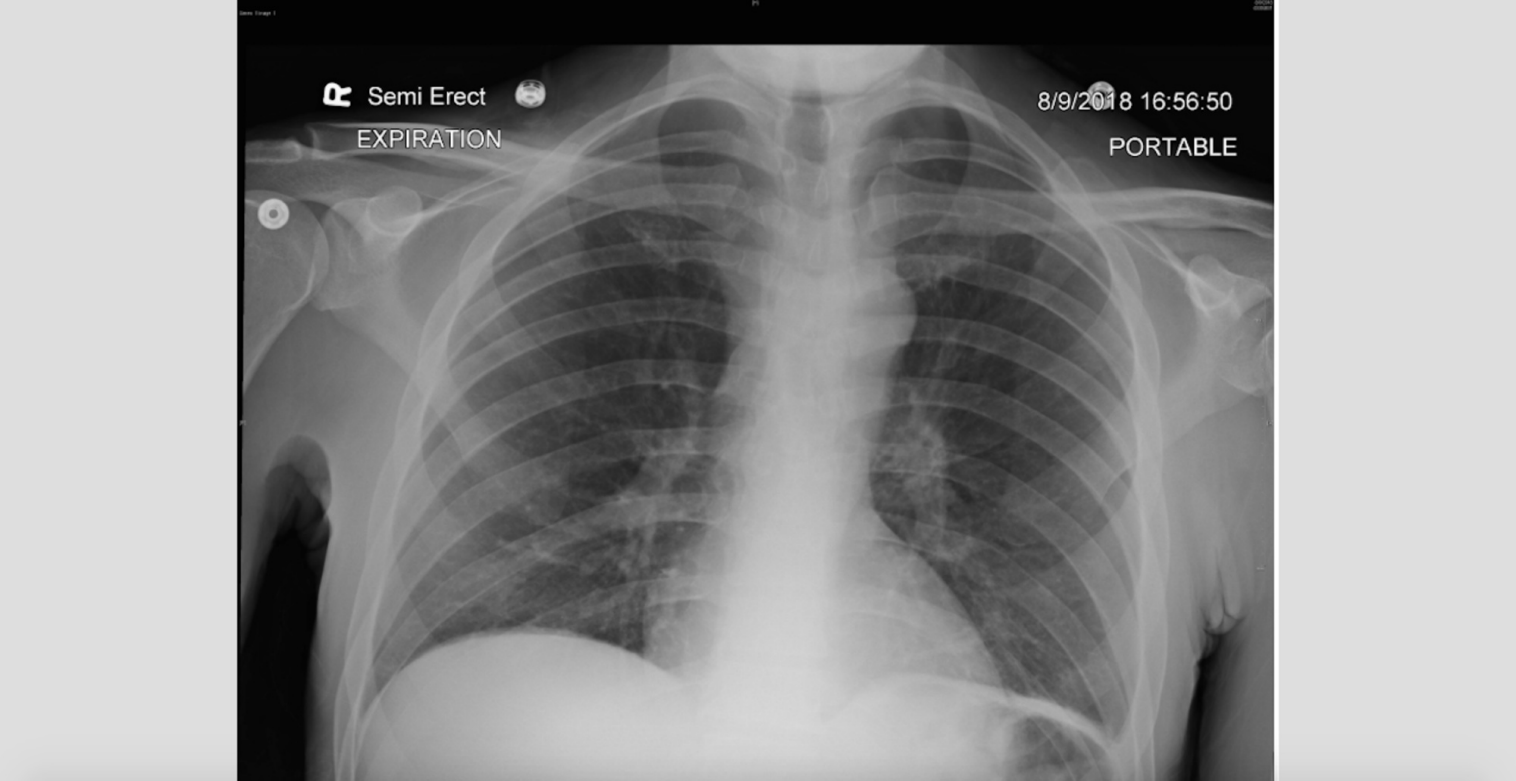
Repeat chest X-ray (CXR) on expiration showing likely spontaneous pneumothorax in the apical upper lobes.

On hospital day four, the patient became febrile overnight with a temperature of 101.5 F. An infectious workup was begun by ordering a chest X-ray, urine and blood cultures. The X-ray showed a questionable pneumothorax at which point radiology recommended a repeat on expiration (Figure 1). The repeat showed what appeared to be a spontaneous pleural bleb rupture and the surgery department was consulted. As the patient was asymptomatic, the surgery department recommended withholding treatment at this time. The cultures were negative.

Over the next few days, the rheumatoid factor came back high at 52 and anti-cyclic citrullinated peptide (anti-CCP) came back >250 confirming inflammatory RA as the definitive diagnosis. The patient was then discharged and told to follow up as an outpatient with the rheumatologist to establish a more definitive treatment plan.

## Discussion

Rheumatoid arthritis has a worldwide distribution with an estimated prevalence of approximately 2%. The prevalence increases with age, approaching nearly 5% in women over age 50. Both the incidence and prevalence are two to three times greater in women compared to men. Although rheumatoid arthritis may present at any age, patients most commonly are first affected in the fourth to sixth decades of life.

In RA, synovial hypertrophy typically leads to chronic joint inflammation known as a “pannus” formation. In very rare instances the inflammation is so severe that it can lead to a loss of cell mass, known as rheumatoid cachexia. Naturally, muscle weakness and a loss of functional capacity is the harbinger of rheumatoid cachexia and is believed to accelerate morbidity and mortality. Currently there is no established definitive mechanism for rheumatoid cachexia, but it is related to an accelerated whole-body protein catabolism and excess production of the inflammatory cytokines such as tumor necrosis  factor-α and interleukin-1β. Tumor necrosis factor-α is probably the central mediator of muscle wasting in rheumatoid arthritis and is known to act synergistically with interleukin-1β in the promotion of cachexia [2]. One would expect this phenomenon to occur in more advanced long lasting RA, however, our case suggests otherwise.

Roughly up to 40% of patients with RA present with extra-articular manifestations. They include the skin, renal, pulmonary, cardiovascular, nervous, and vascular systems. Increased frequency of RF and anti-CCP is correlated with these manifestations. Perhaps severe systemic inflammation brings about these manifestations as well. Pulmonary rheumatoid nodules, including rheumatoid pneumoconiosis (Caplan’s syndrome), although rare, can result in spontaneous pneumothorax and is certainly something to be aware of in an RA patient [3].

## Conclusions

The content of this manuscript serves to enlighten hospitalist physicians and residents about the odd way in which severe RA may present. In the majority of cases, the inflammatory reaction begins with the body's small joints; one should now be aware that rheumatoid arthritis can present itself initially as a large joint disease. Also, most patients with RA have an onset insidious in nature, taking months of high inflammatory states to cause destruction. Our case shows this is not the only disease presentation. Additionally, cachexia is a possible, although rare consequence of the disease as our patient lost over 50 pounds in a few weeks. Furthermore, one should be aware of a spontaneous pneumothorax as a possible extra-articular manifestation such as in the case of our patient.

## References

[1] Nepom GT, Byers P, Seyfried C, Healey LA, Wilskey KR, Stage D, Nepom BS (1989). HLA Genes associated with rheumatoid arthritis. Identification of susceptibility alleles using specific oligonucleotide probes. Arthritis Rheum.

[2] Walsmith J, Roubenoff R (2002). Cachexia in rheumatoid arthritis. Int J Cardiol.

[3] Adelman HM, Dupont EL, Flannery MT, Wallach PM (1994). Recurrent pneumothorax in a patient with rheumatoid arthritis. Am J Med Sci.

